# Awareness of Cancer Risk Factors and Its Signs and Symptoms in Northern Tanzania: a Cross-Sectional Survey in the General Population and in People Living with HIV

**DOI:** 10.1007/s13187-019-01513-6

**Published:** 2019-03-27

**Authors:** Oresto Michael Munishi, Valerie McCormack, Bariki Mchome, Glory Mangi, Leah L. Zullig, John Bartlett, Oscar Mapunda, Pilli Nyindo, Theresia Namwai, Charles Muiruri, Frank Kimaro, Francis Karia, Blandina T. Mmbaga

**Affiliations:** 1 School of Social Sciences, Tampere University, Tampere, Finland; 2 Kilimanjaro Christian Medical Center (KCMC), Moshi, Kilimanjaro Tanzania; 3 Kilimanjaro Clinical Research Institute (KCRI), P.O. Box 2236, Moshi, Kilimanjaro Tanzania; 4 International Agency for Cancer Research (IARC), Lyon, France; 5 Kilimanjaro Christian Medical College (KCMUCo), Moshi, Kilimanjaro Tanzania; 6 Mawenzi Regional Hospital, Moshi, Tanzania; 7 Duke University Medical Center, Durham, NC USA

**Keywords:** Community cancer risk awareness

## Abstract

An important component of cancer control programs for the growing burden in sub-Saharan Africa is a population’s awareness of risk factors. Studies thereof have focused on single rather than multiple cancers and carcinogens. During March and April 2015, we undertook a survey to assess awareness of multiple cancer risk factors and symptoms in the Kilimanjaro Region, North Tanzania. General population (*n* = 620) and attendees at HIV care-and-treatment clinics (CTCs) were included (*n* = 207). Participants’ mean age was 43.8 (interquartile range 30–52) years; 58% were female. Awareness of cancer risk was highest for tobacco (90%) and alcoholic spirits (67%), but tended to be lower for infections (41% for HIV (42.2% and 41.4% for CTC and community group, respectively) and 16% for HPV (16.0% and 16.6% for CTC and community group, respectively)), while that of moldy maize and peanuts was 35% for both. Awareness of specific cancer signs and symptoms ranged between 70% and 90%. Awareness of alcohol and tobacco was higher in men than women (odds ratio = 1.82 (1.38, 2.40) and 3.96 (2.14, 7.31), respectively). In relation to cancer treatment, 70% preferred modern medicine and 10% preferred traditional medicine alone. Sixty percent was not aware of any local cancer early detection services. Only 20% had ever been examined for cancer, and of those screened, CTC group was 1.5 times more likely to screen than community participants. Awareness did not differ by age or HIV status. There are good levels of cancer risk factor awareness for certain lifestyle-related carcinogens in Tanzania; however, increased awareness is needed especially for infections and cancer warning symptom both in the general and HIV-positive population, as well as some myths to be dispelled.

## Introduction

Sub-Saharan Africa (SSA) is experiencing an increasing burden of cancer. GLOBOCAN estimates that the risk of getting and dying of cancer in SSA before age 75 is 12.5% and 9.5%, respectively [1]. It is also projected that between 2010 and 2030, this region will have a 77% increase in cancer deaths [2]. As cancer is relatively new in SSA’s disease profile, the full spectrum of cancer control measures needs strengthening—from prevention strategies to accelerated presentation, diagnosis, and treatment. However, due to barriers throughout this process, as in many low- and middle-income countries, cancer is commonly diagnosed at very advanced disease stages in SSA which contributes to low survival rates [3–5].

In the efforts for cancer control, cancer prevention and early diagnosis are central [6, 7]. In the SSA context, the status of and potential for improvements in these two aspects involve both the population and health system, and at the grassroot level, they start with the population’s understanding of cancer. Religious beliefs, culture, and socioeconomic status influence awareness and knowledge of cancer, while health systems are in different states of preparedness for cancer referral, diagnosis, and treatment [4, 8, 9]. In Africa, culture plays a particular role in disease perceptions and health-seeking behaviors [9]. When cancer symptoms are noticed, knowledge of the importance of early detection can be lacking [9–11]. Cancer awareness is important overall, and especially in high-risk individuals such as people living with HIV/AIDS (PLHIV), a prevalent group in many SSA settings with raised risks of several cancers [12–16].

Previous SSA studies of cancer awareness largely focused on single types of cancer or on specific risk factors. For example, cancer awareness has been studied for cervical cancer especially in relation to HPV and its vaccine [10, 11, 17, 18], as well as for breast, prostate [19–21], lung [22], and esophageal cancer [23]. All these studies were conducted in the general population, while others examined cancer patients’ understanding of the causes of their cancer, unveiling beliefs in curses or witchcraft [24, 25]. In terms of cancer risk factors, studies have focused on awareness of tobacco, STDs, HIV, alcohol consumption [26, 27], and presence of high levels of aflatoxin food contamination [28–30]. None of these studies jointly addressed awareness of multiple cancer risk factors in the population, yet this overall risk is most relevant for an all-encompassing cancer control plan. Thus, to address awareness of cancer in a holistic fashion, in the Kilimanjaro Region of Northern Tanzania, we undertook a study in the general population and in PLHIV to examine awareness of a range of cancer risk factors and of early warning symptoms for all cancer types combined.

## Methods

During March and April 2015, we undertook a cross-sectional survey to assess knowledge and awareness of cancer risk factors and cancer signs and symptoms in the seven districts of Kilimanjaro Region (population 1.6 million), Tanzania. The most common cancers at the region’s tertiary hospital are cervical, breast, esophagus, and prostate [31, 32]. The population can avail of a free cancer early detection or diagnostic services at two major hospitals (Mawenzi and the Kilimanjaro Christian Medical Center (KCMC)) in the region’s capital town of Moshi. Cervical examinations (visual inspection with acetic acid) are organized in a specified clinic at KCMC three times weekly. Mammography and breast examination are available upon request of a clinician. For men, prostatic-specific antigen (PSA) is available to symptomatic men presenting to the urology department.

Two groups of participants were targeted for inclusion in the survey. The first group was population-based; households were randomly selected in each district, and when interviewers approached each house, one adult (minimum age 18 years) was invited to participate on the spot. Total of 620 adults participated, with a response rate of 96%. Such high response rates are typical of this population who are highly willing to facilitate research. The second group consisted of day participants at five HIV care-and-treatment clinics (HIV-CTCs), four of which are situated within inpatient and outpatient departments of secondary/tertiary hospitals and one was a stand-alone clinic. HIV-CTCs provide PLHIV with ARVs, treatment monitoring, and checkups. As in the rest of the country, a health education session to clients is a common procedure in most of HIV-CTC within the study area. Lesson of the day may range from a wide selection of topics including but not limited to cancer screening, adherence to treatment, nutritional needs, self-care, etc. HIV-CTC attendees were randomly selected from the patients’ waiting list on clinic days, from which 207 participants were enrolled.

For each participant, written informed consent was obtained prior to conducting interviews using a pre-tested interviewer-administered questionnaire in Kiswahili. For knowledge of cancer risk factors, participants were asked whether each of a list of items is common in this setting “increased cancer risk,” with the answers “yes, a strong cause of cancer,” “yes, a moderate cause of cancer,” “no, not related to cancer,” and “I don’t know.” Fourteen of the items were expected to be answered in either of the “yes” categories [33]: [1] tobacco use, [2] any alcohol, [3] spirits, [4] home brew, [5] EBV, [6] hepatitis B, [7] HIV/AIDS, [8] HPV, [9] indoor air pollution, [10] relative with breast cancer, [11] old age, [12] overweight or obesity, [13] moldy groundnuts as proxy for aflatoxin, and [14] processed meat. If the person identified the carcinogen, they were asked what types of cancer it could cause, with multiple types allowed. Intermixed with these risk factors were other exposures prevalent in the area or likely to be of concern to the community, but that are not established carcinogens at present.

Cancer warning signs/symptoms were obtained from the American Cancer Society seven-cancer warning signs abbreviated as c-a-u-t-i-o-n [34] which range from skin, gastrointestinal, breast, lung, and cervix symptoms. Out of a list of 11 mixed signs and symptoms of ill health, 7 were obvious cancer warning signs. Response options were yes, “no,” and “I don’t know.”

Ethical approval was obtained from the Kilimanjaro Christian Medical University Collage Institutional Review Board (CRERC). We obtained permission to contact the community from regional and district medical officers.

### Statistical Analysis

R-studio^R^ software [35] was used to analyze the descriptive characteristics of the study participants. Univariate and multivariate logistic regressions were used to compute measures of association between cancer awareness as a binary outcome and the explanatory variables age, sex, district of residence, and HIV status. Binary outcome constituted “knowledgeable” which is a correct response to the known cancer risk factors or warning sign, and “non-knowledgeable” is the opposite.

## Results

### Participant Characteristics

Characteristics of the 827 study participants (620 community and 207 HIV-CTC attendees) are shown in Table 1. Overall, 58% were women, especially in PLHIV (67%). The mean age of participants in the community was 41.7 years and 44.2 years in the HIV group.

**Table 1:** Characteristics of study respondents, by recruitment place

*N* (%)		HIV care-and-treatment clinic (*N* = 207)	Community *N* = 620
Gender	Male	68 (33)	283 (47)
	Female	139 (67)	337 (63)
Districts	Moshi urban	31 (15)	73 (12)
	Moshi rural	31 (15)	99 (16)
	Hai	29 (14)	82 (13)
	Siha	30 (15)	90 (14)
	Rombo	25 (12)	97 (16)
	Mwanga	31 (15)	84 (14)
	Same	30 (14.5)	96 (15)
Age (years)	Median (IQR)	44 (36–53)	40 (30–52)

### Awareness of Risk Factors for Cancer

**Fig. 1:**
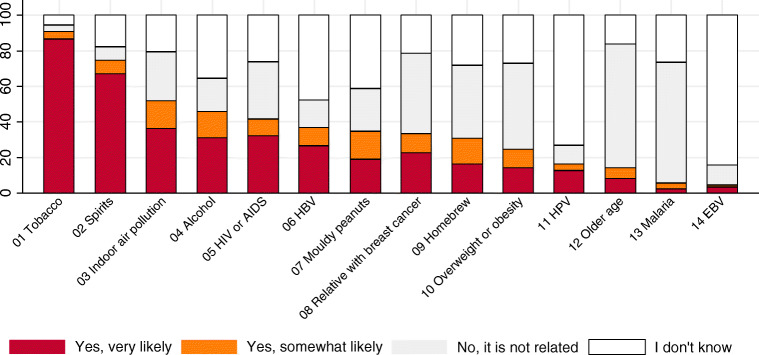
Awareness of cancer risk factors, for established cancer risk factors at the time of the study

Figure 1 shows the awareness distribution of each cancer risk factor. Awareness levels were highest for tobacco (90%). For consumption of any alcohol type, awareness of cancer risk was 46%. Cancer risk awareness differed greatly between types of alcohol. It was lowest for home-brewed alcohol at 16% (*n* = 132) and highest for strong sprits, 67% (*n* = 554), for which 54% (*n* = 334) of affirmatives said it caused liver cancer, 20% (*n* = 124) esophageal cancer, 19% (*n* = 117) lung cancer, and 12.7% (*n* = 79) gastric cancer. Amongst those who answered affirmatively, cancer types reported as being related to tobacco were lung 72% (*n* = 541) and esophageal 32% (*n* = 240).

Awareness of infections related to cancer was generally lower: 41% (*N* = 267) of participants knew that HIV increases risk of developing cancer, 28% (*n* = 96) of positive responders related it to cervical cancer, and 19% (*n* = 65) listed skin cancer. When analyzed separately by HIV or community participants, there was no difference in those who know HIV was associated with cancer risk. Knowledge of cancer risks associated with HPV and HBC was lower at 17% (*n* = 136) and 37% (*n* = 305), respectively. Awareness of risk associated with moldy maize or peanut was both 35% (*n* = 287) while awareness of indoor air pollution was 38% (*n* = 429). Knowledge of risk of being overweight was 24% (*n* = 203), and that of processed meat consumption was 38% (*n* = 317). Of other agents queried that are not recognized cancer risk factors (data not shown in tables), mobile phones were of concern to participants, either kept in the pocket or when using it (both > 50%) and use of pesticides (67%) (Table 2).

**Table 2:** Distribution of awareness of cancer risk factors and odds ratios for awareness by demographic factors and HIV status

Agent	Response to “Does [agent] cause cancer” *N* (%)				Most commonly mentioned cancer types** (% of participants who responded “yes” who mentioned this cancer)	Odds ratio (95% CI) of risk factor awareness (“yes very likely” or “yes, somewhat likely” vs answer “no” or “I do not know”) associated with:		
	Yes, very likely	Yes, somewhat likely	No, it’s not related	I do not know		HIV+ vs HIV−	Sex Male vs female	Age ≥ 50 vs < 50 years
Environmental, lifestyle, and genetic								
Tobacco use	715 (86)	37 (4)	29 (4)	47 (7)	Lung (72), esophagus [32]	0.80 (0.47, 1.35)	3.96 (2.14, 7.31)	1.38 (0.80, 2.38)
Drinking alcohol	258 (31)	122 (15)	153 (18)	295 (36)	Liver [30], esophagus [24], gastric [24]	0.79 (0.58, 1.09)	1.82 (1.38, 2.40)	0.85 (0.63, 1.14)
Home-brewed alcohol	135 (16)	120 (14)	340 (41)	233 (28)	Liver [29], stomach [31], esophagus [15]	1.95 (1.41, 2.71)	1.2 (0.89, 1.62)	1.25 (0.91, 1.71)
Strong sprits	555 (67)	64 (8)	62 (7)	147 (18)	Liver (54), esophagus [20]	0.80 (0.56, 1.14)	1.65 (1.19, 2.29)	1.08 (0.77, 1.53)
Indoor air pollution	300 (36)	129 (2	228 28)	171 (21)	Lung (72), esophagus [13]	0.99 (0.73, 1.36)	1.13 (0.86, 1.49)	1.11 (0.82, 1.49)
Moldy peanuts	155 (19)	131 (16)	200 (24)	342 (41)	Stomach (72), esophagus [8], liver [4]	1.34 (0.97, 1.86)	1.21 (0.91, 1.62)	1.31 (0.96, 1.77)
Moldy maize	157 (19)	130 (16)	198 (24)	343 (41)	Stomach (70), esophagus [8]	1.3 (0.94, 1.79)	1.23 (0.92, 1.64)	1.43 (1.05, 1.94)
Processed meat	172 (21)	145 (17)	168 (20)	342 (41)	Stomach (53), blood [7]	0.69 (0.49, 0.96)	1.13 (0.85, 1.50)	0.91 (0.67, 1.23)
Relative with breast cancer	188 (23)	88 (11)	375 (45)	177 (21)	Breast (75)	0.97 (0.70, 1.36)	1.22 (0.91, 1.63)	1.00 (0.73, 1.36)
Overweight, obesity	118 (14)	85 (10)	399 (48)	226 27	Liver [22], stomach [36] esophagus [16]	1.2 (0.84, 1.71)	1.14 (0.83, 1.57)	1.1 (0.78, 1.55)
Infections								
HIV	267 (32)	76 (9)	266 (32)	219 (26)	Cervical [28], skin [19]	1.03 (0.75, 1.42)	1.03 (0.78, 1.37)	0.68 (0.50, 0.92)
EBV	27 (3)	10 (1)	95 (11)	696 (84)	Cervical (60)	0.82 (0.37, 1.82)	2.32 (1.18, 4.57)	0.6 (0.27, 1.34)
HBV	220 (27)	85 (10)	129 (16)	394 (48)	Liver (78)	1.02 (0.74, 1.41)	0.75 (0.56, 1.00)	1.02 (0.75, 1.39)
HPV	104 (13)	32 (4)	85 10)	607 (73)	Cervical (79)	0.95 (0.62, 1.46)	1.09 (0.75, 1.58)	1.05 (0.71, 1.56)

The combined percentage exceeds 100% because one person could mention more than one cancer for each risk factor

Associations with cancer risk factor awareness generally found few differences by age, sex, or HIV status with exception of the following. We found a statistically significant gender differences in alcohol risk awareness, overall and for strong spirits. Men were more knowledgeable than women of the cancer risks posed by alcohol (OR 1.8 (95% confidence interval 1.25–2.26), *p* = 0.0006) and by strong sprits (OR 1.65 (95% CI 1.19–2.29), *p* = 0.0002). Similar to alcohol, knowledge on the risk of tobacco use was higher in men than women (OR 3.96 (95% CI 2.14–7.31), *p* = 0.001). Knowledge of infection risk of HPV and HIV infection did not show any significant relation to age or sex of respondents, but knowledge to risk of Epstein Barr virus was higher for men (OR 2.32 (95% CI 1.18–4.57), *p* = 0.001). HIV-positive people (CTC group) had greater awareness of risks posed by homebrew (OR = 1.95 (CI = 1.41–2.71), *p* = 0.004), but were less knowledgeable of risks of processed meat (OR = 0.69 (CI = 0.48–0.96), *p* = 0.001); however, such meats are not commonly consumed in this setting. Older people (> 50 years) were more knowledgeable to risk of moldy maize (OR = 1.43 (CI = 1.5–1.94), *p* = 0.0001) and less knowledgeable to risk of HIV infection (OR = 0.68 (CI = 0.50–0.92), *p* = 0.0003).

### Knowledge of Cancer Warning Symptoms

The majority of respondents, 71% (*n* = 587), said that an obvious growth, rough, or blackening skin spot could be a warning sign of cancer. Similarly, 74% (*n* = 612) said yes for an obvious change in a wart or mole, 77% (*n* = 636) identified a sore throat that does not heal, 71 (*n* = 587) unusual bleeding or discharge, and 90% (*n* = 744) a thickening or lump in the breast or elsewhere in body. Other signs/symptoms of cancer and their community awareness levels are indicated in Table 3. Mutually adjusted odds ratios of sign and symptom awareness only demonstrated differences for awareness of the unusual bleeding and discharge, which was higher for older respondents (> 50 years) (OR = 1.46 (CI = 1.05–2.05), *p* = 0.003). HIV status and sex did not have any influence on knowledge of cancer warning signs/symptoms.

**Table 3:** Knowledge of cancer warning signs and symptoms

Likely cancer warning symptom, *N* (%)	Yes cancer warning symptom		Not cancer warning symptom		I do not know		Odds ratio of being aware of the cancer sign or symptom		
Cancer warning symptoms	HIV-CTC	Community	HIV-CTC	Community	HIV-CTC	Community	HIV+ vs HIV−	Sex male vs female	Age ≥ 50 vs < 50 years
Change of bowel or bladder habits	93 (45)	232 (37)	87 (42)	262 (42)	27 (13)	127 (20)	0.8 (0.58, 1.11)	1.19 (0.89, 1.57)	0.87 (0.65, 1.19)
Unusual bleeding or discharge	147 (71)	437 (70)	42 (20)	99 (16)	18 (8)	95 (15)	0.91 (0.65, 1.28)	1.28 (0.95, 1.74)	1.46 (1.05, 2.05)
Thickening or lump in breast/elsewhere	193 (94)	552 (88)	7 (3)	28 (5)	7 (3)	41 (7)	1.14 (0.66, 1.95)	1.34 (0.84, 2.14)	1.04 (0.64, 1.71)
Indigestion or difficult in swallowing	124 (60)	382 (21)	56 (27)	156 (25)	27 (13)	83 (13)	1.16 (0.84, 1.61)	0.99 (0.75, 1.31)	1.2 (0.89, 1.63)
Obvious change in a wart or mole	154 (74)	458 (73)	31 (18)	102 (16)	16 (8)	61 (10)	1.03 (0.72, 1.48)	1.01 (0.74, 1.39)	0.89 (0.64, 1.24)
Nagging cough or hoarseness	115 (56)	363 (58)	66 (32)	168 (27)	26 (13)	90 (14)	1.36 (0.98, 1.88)	1.03 (0.78, 1.36)	1.03 (0.77, 1.39)
A sore throat that does not heal	165 (80)	447 (76)	25 (12)	88 (15)	17 (8)	56 (9)	0.95 (0.65, 1.38)	1.08 (0.78, 1.51)	1.2 (0.84, 1.73)

*HIV CTC* HIV care-and-treatment clinic participants

### Knowledge of Cancer Early Detection and Treatment Options

When respondents were asked what treatment option they would choose if themselves or their relatives had cancer, 10% (*n* = 83) preferred traditional medicine while 20% (*n* = 166) preferred a combination of both traditional and modern medicine. The rest (70%) preferred modern medicine. Further, we found that 58% (*n* = 480) of people interviewed were not aware of cancer early detection services in Moshi. Awareness was not influenced by HIV status, sex, or age of the respondent. When asked about previous cancer screening, only 18% (*n* = 63) of men and 20% (*n* = 95) of women had ever screened for cancer before. However, HIV-CTC respondent was one and a half times more likely to screen for cancer than the household respondents (OR = 1.58 (1.07–2.31), *p* = 0.02). The majority of screened cancers by participants were cervix 75% (*n* = 114) and breast 20% (*n* = 33). Two hundred twenty (71%) of participants who responded mentioned KCMC, and 7% mentioned Mawenzi as available cancer care sites in Moshi (Table 4).

**Table 4:** Awareness of cancer care and treatment options in Kilimanjaro

Views on cancer service opinions and uptake, *N* (%)	HIV-CTC	Community	HIV-CTC	Community	HIV-CTC	Community	Odds ratio of being aware of local cancer services and uptake		
	Yes		No		I do not know		HIV+ vs HIV−	Sex male vs female	Age ≥ 50 vs < 50 years
Do you know any cancer diagnosis service in Kili?	64 (31)	229 (37)	132 (64)	347 (56)	11 (5)	45 (7)	0.96 (0.68–1.34)	0.99 (0.74–1.33)	1.02 (0.75–1.39
Have you ever been screened for any cancer?	37 (18)	119 (19)	168 (81)	498 (80)	2 (1)	4 (1)	1.58 (1.07–2.31)	0.95 (0.67–1.37)	0.82 (0.55–1.20)
Do you know any cancer care/treatment center in kili?	70 (34)	241 (39)	128 (62)	343 (55)	9 (4)	35 (6)	0.87 (0.62–1.21)	1.12 (0.84–1.50)	0.90 (0.66–1.22)
	Modern		Traditional		Both				
If yourself or relative had cancer, which care/treatment option would you choose	142 (69)	433 (70)	23 (11)	63 (10)	41 (40)	125 (20)			

HH = community participants. CTC = HIV care-and-treatment clinic participants

## Discussion

Awareness of cancer risk factors and early symptoms is important for prevention and early detection of cancer [18, 37]. A community’s knowledge of risk factors helps individuals take personal preventive actions in the avoidance of carcinogenic lifestyle habits. Additionally, awareness of cancer early warning symptoms and the understanding of the potential for curative or palliative treatment influence time to presentation and diagnosis. In the present study, we examined cancer awareness in the general population and HIV-positive population of Northern Tanzania, which were generally high for alcohol and tobacco in particular. We found that 75% of people understood the cancer risks linked to strong spirits—particularly men who tend to be the consumers of this type of alcohol in this community. Even though only a small proportion of people thought that the well-known and highly consumed home brew called “*mbege*” was a risk for any cancer, still the general awareness on risk of alcohol is good. As found out by Dong et al. [36] and discussed in the paper by McCormack et al. [38], awareness of risk posed by strong spirits [36, 39] is particularly important in this setting where esophageal cancer and other gastrointestinal malignancies are common. In the Kilimanjaro Region where this study was conducted, this is even more important because of the recent insurgency of industrially brewed strong alcohol locally called “*kiroba*”—spirits sold in small plastic sachets—very popular among the youth [38]. Similarly, awareness of tobacco use was generally high, and though it was lower in women, they are less likely to be tobacco users, so this lack of awareness may not be critical. On the other hand, women are key educators of children; thus, filling this awareness gap is still needed for the health of future generations. These awareness levels were similar to those found in other studies [27, 36]. In comparison with a similar survey by the American Institute for Cancer Research in 2015 [40], risk factor awareness in the US general population differed by small margin from our findings. In 2015, awareness of several cancer risks was tobacco (94%), viruses and bacteria (55%), overweight (52%), alcohol (43%), pesticide residues (74%), cured meat (38%), and genetic predisposition (89%).

For more than two decades, HIV care programs in Tanzania have provided clinic-based health education to its clients aimed at improving prevention against opportunistic infections and other illnesses including cancer [41, 42]. Included in the HIV-CTC health education message is information on increased risk for cervical cancer for HIV-positive women. In many HIV-CTCs, there is a referral pathway for at-risk women to access cancer screening services usually offered in annexes of major hospital(s) in the region. Consequently, we found that HIV-CTC participants had one and half times more likelihood of attending cancer early detection programs than the household participants, indicating successful education programs in this group. Although this study did not assess in detail the composition of messaging given at the sessions, we believe that this difference in screening uptake is a result of the health education provided at CTC.

Nevertheless, knowledge of infection-related cancer risks among HIV+ people did not differ except for home-brewed alcohols and processed meat (where their awareness was higher). The lack of difference in cancer risk knowledge between these two groups is however of concern, and the effectiveness of education programs in HIV+ people on cancer risk factors and symptom awareness needs strengthening. The reasons for higher screening uptake but low awareness of cancer risk posed by infections such as HIV need to be addressed. This may have resulted from selective uptake of messages from health information packages. This calls for analysis and possible review of content and structure of the health education messages to determine factors influencing its uptake by the target population. This study did not assess the education level of participants; however, a report by UNESCO [43] pointed out that in 2012, literacy rates for persons 15 years and above had risen to 92% with more literate men than women (90% and 94%, respectively). About 83% had primary education and up to 13% with secondary education.

Certain factors were considered as cancer risk factors by this Tanzanian community, yet they are not established carcinogens. With the majority of the population as small-scale farmers with substantial fertilizer use, the question of pesticide use is important for these residents. Use of certain pesticides, DDT (dichlorodiphenyltrichloroethane) in particular, has been investigated in relation to cancer, but is a probable and not a confirmed carcinogen (group 2A (updated in 2017 [33, 44, 45]. In Kilimanjaro, pesticides have been used for more than six decades on coffee plantations and recently opened horticulture farms. The extensive use of these chemicals seems to startle the community. Sixty-four percent thought there is high risk for cancer associated with pesticide use. Mobile phone use was also of concern to individuals, with many considering it as being associated with cancer. In 2013, IARC classified use of wireless phone as possibly carcinogenic (group 2B) [46]. Even if agents are not carcinogenic, at times, there may be needs to specifically address these fears in the local population or to dispel myths of misheld beliefs.

Signs and symptoms can be a good driving factor for early patient presentation. As discussed by Ngoma and Wamai et al. [18, 37], external lesions are important driving factors for health care–seeking behavior. If awareness was raised selectively on early skin manifestation, majority of cancer cases would be diagnosed while still in a curable stage. In this survey, majority of respondents (90%) said that an obvious growing skin lump could be a warning sign as it was for unusual bleeding and discharge (71%). Awareness to other signs such as breast lumps, hoarseness, and difficulty swallowing could potentially downstage cancer diagnosis in this community.

Although organized cancer screening is not recommended, and because Africa is yet to establish customized African code against cancer as that for the European countries [47], it is recommended that local practices as detailed in the National Cancer Control Strategy (NCCS 2013–2022) for Tanzania, strategic objective 2.4 [48] be followed. This in addition to many other things; it encourages development and dissemination of educational material on the importance of screening and early detection targeted to the entire population with more emphasis to rural and vulnerable groups. Despite evidence of reduction [49, 50] of cancer risk to HIV patients on combination anti-retroviral drugs (cART), cancer screening or early detection among HIV and especially immunocompromised HIV patients should be encouraged. This should be included as part of discussion topics in the HIV-CTC health education package. HIV-positive women should be encouraged to screen for cervical cancer as per guidelines and NCCS-2013-2022 [48, 51]. The fact that 35% of all women knew about early detection services but only 20% had ever attended early detection is of concern. For these early detection programs to be effective and sustainable, awareness of care points is important. Our survey found that KCMC Hospital was widely known for its early detection and other cancer care services. However, other cancer care centers (such as Mawenzi Hospital) were not as known despite the presence of similar services. Information on care centers needs to be communicated effectively.

As far as we know, this is the first study in Kilimanjaro and Tanzania to ever investigate knowledge and collective awareness of cancer risks mediated by individual agents. Improved diagnosis has raised the public awareness and debate on potential cancer risk exposures. Much as this could mean increased attention to cancer signs and symptoms by clinicians and hence improved diagnosis, many people in the community perceive this as an increase in cancer incidence, and thus, they look to attribute this apparent increase in recent changes in their lives such as methods of food production, processing, and meal preparations. While some of these hypotheses may be true, further research on known and perceived cancer risks of environmental relevance is needed. Results from our study will therefore inform local cancer prevention programs and will set a benchmark for further risk awareness research.

In conclusion, knowledge and awareness to cancer risks and cancer warning symptoms in Tanzania are high and diversified. While people seem to be highly aware to several risks to cancer especially those pertaining to alcohol and smoking, they are also significantly less aware about some other agents that have the same carcinogenic potentials such as HIV infection. The observed difference could be due to selective awareness raised by anti-smoking campaigns. More awareness is needed for particularly other agents that could potentially expose people to the risks of developing cancer.

## References

[1] Globocan 2012: Estimated cancer incidence mapw. (2012) Globocan

[2] Rebbeck TR (2013). Handbook for cancer research in Africa.

[3] Ali-Risasi C, Mulumba P, Verdonck K, Vanden Broeck D, Praet M (2014). Knowledge, attitude and practice about cancer of the uterine cervix among women living in Kinshasa, the Democratic Republic of Congo. BMC Womens Health.

[4] Sudenga SL, Rositch AF, Otieno WA, Smith JS (2013). Knowledge, attitudes, practices, and perceived risk of cervical cancer among Kenyan women: brief report. Int J Gynecol Cancer.

[5] WHO (2014). World cancer report 2014. 2014 ed.

[6] Nieburg HE (2005). Cancer prevention & control strategy resolution adopted by the 58th World Health Assembly, Geneva, May 25, 2005. Cancer Detect Prev.

[7] Verma M (2013). Cancer control and prevention: nutrition and epigenetics. Curr Opin Clin Nutr Metab Care.

[8] McCaffery K, Wardle J, Waller J (2003). Knowledge, attitudes, and behavioral intentions in relation to the early detection of colorectal cancer in the United Kingdom. Prev Med.

[9] van Schalkwyk SL, Maree JE, Dreyer Wright SC (2008). Cervical cancer: the route from signs and symptoms to treatment in South Africa. Reproductive Health Matters.

[10] Mwaka AD, Okello ES, Kiguli J, Rutebemberwa E (2014). Understanding cervical cancer: an exploration of lay perceptions, beliefs and knowledge about cervical cancer among the Acholi in northern Uganda. BMC Womens Health.

[11] Mwaka AD, Orach CG, Were EM, Lyratzopoulos G, Wabinga H, Roland M (2016). Awareness of cervical cancer risk factors and symptoms: cross-sectional community survey in post-conflict northern Uganda. Health Expect : Int J Public Participation Health Care Health Policy.

[12] Aserlind A, Maguire K, Duthely L, Wennin S, Potter J. (2017) Women living with HIV over age of 65: cervical cancer screening in a unique and growing population. Infect Dis Obstet Gynecol 2017: 2105061, 1, 5 10.1155/2017/2105061 PMC5623791 29075090

[13] Brotherton JML, Giuliano AR, Markowitz LE, Dunne EF, Ogilvie GS (2016). Monitoring the impact of HPV vaccine in males—considerations and challenges. Papillomavirus Res.

[14] Mendes D, Mesher D, Pista A, Baguelin M, Jit M (2017) Understanding differences in cervical cancer incidence in Western Europe: comparing Portugal and England. Eur J Pub Health 10.1093/eurpub/ckx176 29059348

[15] Obiri-Yeboah D, Akakpo PK, Mutocheluh M, Adjei-Danso E, Allornuvor G, Amoako-Sakyi D, Adu-Sarkodie Y, Mayaud P (2017). Epidemiology of cervical human papillomavirus (HPV) infection and squamous intraepithelial lesions (SIL) among a cohort of HIV-infected and uninfected Ghanaian women. BMC Cancer.

[16] Wanyenze RK, Bwanika JB, Beyeza-Kashesya J, Mugerwa S, Arinaitwe J, Matovu JKB, Gwokyalya V, Kasozi D, Bukenya J, Makumbi F (2017). Uptake and correlates of cervical cancer screening among HIV-infected women attending HIV care in Uganda. Glob Health Action.

[17] Birhanu Z, Abdissa A, Belachew T, Deribew A, Segni H, Tsu V, Mulholland K, Russell FM (2012). Health seeking behavior for cervical cancer in Ethiopia: a qualitative study. Int J Equity Health.

[18] Wamai RG, Ayissi CA, Oduwo GO, Perlman S, Welty E, Manga S, Ogembo JG (2012). Assessing the effectiveness of a community-based sensitization strategy in creating awareness about HPV, cervical cancer and HPV vaccine among parents in North West Cameroon. J Community Health.

[19] Muliira JK, Al-Saidi HS, Al-Yahyai AN (2017). Determinants of behavioral intentions to screen for prostate cancer in Omani men. Asia Pac J Oncol Nurs.

[20] Ojewola RW, Oridota ES, Balogun OS (2017). Knowledge, attitudes and screening practices regarding prostatic diseases among men older than 40 years: a population-based study in Southwest Nigeria. Pan Afr Med J.

[21] Rivas C, Matheson L, Nayoan J, Glaser A, Gavin A, Wright P, Wagland R, Watson E (2016). Ethnicity and the prostate cancer experience: a qualitative metasynthesis. Psychooncology..

[22] Desalu OO, Fawibe AE, Sanya EO, Ojuawo OB, Aladesanmi AO, Salami AK (2016). Lung cancer awareness and anticipated delay before seeking medical help in the middle-belt population of Nigeria. Int J Tuberc Lung Dis.

[23] Duron V, Bii J, Mutai R, Ngetich J, Harrington D, Parker R, White R (2013). Esophageal cancer awareness in Bomet district, Kenya. Afr Health Sci.

[24] Ayanniyi AA, Jamda AM, Badmos KB, Adelaiye RS, Mahmoud AO, Kyari F, Nwana EJ (2010). Awareness and knowledge of ocular cancers in a resource-limited economy. N Am J Med Sci.

[25] Naanyu V, Asirwa CF, Wachira J, Busakhala N, Kisuya J, Otieno G, Keter A, Mwangi A, Omenge OE, Inui T (2015). Lay perceptions of breast cancer in Western Kenya. World J Clin Oncol.

[26] Faggons CE, Mabedi CE, Liomba NG, Funkhouser WK, Chimzimu F, Kampani C, Krysiak R, Msiska N, Shores CG, Gopal S (2017). Human papillomavirus in head and neck squamous cell carcinoma: a descriptive study of histologically confirmed cases at Kamuzu Central Hospital in Lilongwe, Malawi. Malawi Med J.

[27] Loots E, Sartorius B, Madiba TE, Mulder CJJ, Clarke DL (2017). Oesophageal squamous cell cancer in a South African tertiary hospital: a risk factor and presentation analysis. S Afr J Surg.

[28] Seetha A, Munthali W, Msere HW, Swai E, Muzanila Y, Sichone E, Tsusaka TW, Rathore A, Okori P (2017). Occurrence of aflatoxins and its management in diverse cropping systems of central Tanzania. Mycotoxin Res.

[29] Mmongoyo JA, Wu F, Linz JE, Nair MG, Mugula JK, Tempelman RJ, Strasburg GM (2017). Aflatoxin levels in sunflower seeds and cakes collected from micro- and small-scale sunflower oil processors in Tanzania. PLoS One.

[30] Kamala A, Kimanya M, Lachat C, Jacxsens L, Haesaert G, Kolsteren P, Ortiz J, Tiisekwa B, de Meulenaer B (2017). Risk of exposure to multiple mycotoxins from maize-based complementary foods in Tanzania. J Agric Food Chem.

[31] Ferlay J, Soerjomataram I, Dikshit R, Eser S, Mathers C, Rebelo M, Parkin DM, Forman D, Bray F (2015). Cancer incidence and mortality worldwide: sources, methods and major patterns in GLOBOCAN 2012. Int J Cancer.

[32] Hiza PR (1976). Malignant disease in Tanzania. East Afr Med J.

[33] IARC (2017). IARC monographs on the evaluation of carcinogenic risks to human.

[34] Partners in Health W (2016). The seven warning signs of cancer.

[35] R software. (2016) R. statistical software. R. Pp. R.Statisical software

[36] Dong J, Thrift AP (2017). Alcohol, smoking and risk of oesophago-gastric cancer. Best Pract Res Clin Gastroenterol.

[37] Ngoma T, Mandeli J, Holland JF (2015). Downstaging cancer in rural Africa. Int J Cancer.

[38] McCormack VA, Menya D, Munishi MO (2017). Informing etiologic research priorities for squamous cell esophageal cancer in Africa: a review of setting-specific exposures to known and putative risk factors. Int J Cancer.

[39] Na H-K, Lee J (2017). Molecular basis of alcohol-related gastric and colon cancer. Int J Mol Sci.

[40] (AICR) AIfCR. (2015) Cancer awarenes report. 2015

[41] van der Kop ML, Muhula S, Nagide PI et al (2018) Effect of an interactive text-messaging service on patient retention during the first year of HIV care in Kenya (WelTel Retain): an open-label, randomised parallel-group study. Lancet Public Health 10.1016/S2468-2667(17)30239-6 PMC5884138 29361433

[42] DiCarlo A, Fayorsey R, Syengo M, Chege D, Sirengo M, Reidy W, Otieno J, Omoto J, Hawken MP, Abrams EJ (2018). Lay health worker experiences administering a multi-level combination intervention to improve PMTCT retention. BMC Health Serv Res.

[43] UNESCO. (2014) Education for all (EFA) report for Tanzania mainland:2014. Nov 2014 ed.

[44] Bornman M, Delport R, Farias P (2018). Alterations in male reproductive hormones in relation to environmental DDT exposure. Environ Int.

[45] Alleva R, Manzella N, Gaetani S, Bacchetti T, Bracci M, Ciarapica V, Monaco F, Borghi B, Amati M, Ferretti G, Tomasetti M (2018). Mechanism underlying the effect of long-term exposure to low dose of pesticides on DNA integrity. Environ Toxicol.

[46] (IARC) IAfRoC. (2013) Non-ionizing radition part 2: radiofrequency electromagnetic fields. IARC PMC4780878 24772662

[47] Espina. (2018) European code against cancer. IARC information 2018

[48] MoH. (2013) Tanzania National Cancer Control Strategy (2013–2022)

[49] Borges ÁH (2017). Combination antiretroviral therapy and cancer risk. Curr Opin HIV AIDS.

[50] Chun C, Leyden WA, Lanfang X (2012). Exposure to antiretroviral therapy and risk of cancer in HIV-infected persons. AIDS (London, England).

[51] Virani S, Sriplung H, Bilheem S, Sripan P, Maneesai P, Waisri N, Chitapanarux I (2018). Effect of the national screening program on malignancy status of cervical cancer in northern Thailand. Int J Public Health.

